# Default Mode Network as a Neural Substrate of Acupuncture: Evidence, Challenges and Strategy

**DOI:** 10.3389/fnins.2019.00100

**Published:** 2019-02-11

**Authors:** Yuqi Zhang, Haolin Zhang, Till Nierhaus, Daniel Pach, Claudia M. Witt, Ming Yi

**Affiliations:** ^1^Department of Neurobiology, School of Basic Medical Sciences, Neuroscience Research Institute, Peking University, Beijing, China; ^2^Department of Traditional Chinese Medicine, Peking University Third Hospital, Beijing, China; ^3^Neurocomputation and Neuroimaging Unit, Department of Education and Psychology, Freie Universität Berlin, Berlin, Germany; ^4^Department of Neurology, Max Planck Institute for Human Cognitive and Brain Sciences, Leipzig, Germany; ^5^Institute for Social Medicine, Epidemiology, and Health Economics, Charité-Universitätsmedizin Berlin, Corporate Member of Freie Universität Berlin, Humboldt-Universität zu Berlin, and Berlin Institute of Health, Berlin, Germany; ^6^Institute for Complementary and Integrative Medicine, University Hospital Zurich, University of Zurich, Zurich, Switzerland; ^7^Center for Integrative Medicine, University of Maryland School of Medicine, Baltimore, MD, United States; ^8^Key Laboratory for Neuroscience, Ministry of Education, National Health Commission, Peking University, Beijing, China

**Keywords:** acupuncture, default mode network, neuroimaging, pain, fMRI

## Abstract

Acupuncture is widely applied all over the world. Although the neurobiological underpinnings of acupuncture still remain unclear, accumulating evidence indicates significant alteration of brain activities in response to acupuncture. In particular, activities of brain regions in the default mode network (DMN) are modulated by acupuncture. DMN is crucial for maintaining physiological homeostasis and its functional architecture becomes disrupted in various disorders. But how acupuncture modulates brain functions and whether such modulation constitutes core mechanisms of acupuncture treatment are far from clear. This *Perspective* integrates recent literature on interactions between acupuncture and functional networks including the DMN, and proposes a back-translational research strategy to elucidate brain mechanisms of acupuncture treatment.

## Complex Brain Mechanisms of Acupuncture

Acupuncture, an important component of traditional Chinese medicine, has been practiced in China for more than 3000 years, and is now widely applied all over the world (Zhuang et al., 2013). Studies have shown that for disorders such as chronic pain, the effects of acupuncture cannot be fully attributed to placebo (Vickers et al., 2012, 2018). Neuroimaging studies have revealed significant brain activity changes in response to acupuncture, indicating possible brain contribution to its effects.

Intriguingly, brain responses to acupuncture stimuli encompass a broad network of regions involving not only somatosensory, but affective and cognitive processing. A meta-analysis of brain activities associated with acupuncture stimulation reveals activation in the sensorimotor cortical network, including the insula, thalamus, anterior cingulate cortex (ACC), and primary and secondary somatosensory cortices, and deactivation in the limbic-paralimbic neocortical network, including the medial prefrontal cortex (mPFC), caudate, amygdala, posterior cingulate cortex (PCC), and parahippocampus (Chae et al., 2013). These findings indicate multi-dimensional brain responses to acupuncture. However, contribution of each dimension to acupuncture effects is poorly defined.

Additional complexity stems from differences between various acupuncture paradigms (Huang et al., 2012). Such variations may stem from (but not restricted to) manual versus electro-acupuncture, electro-acupuncture of different stimulating frequencies and intensities, acupuncture in different points, responders versus non-responders of acupuncture, and acupuncture in healthy versus morbid participants (Han, 2003; Yi et al., 2011; Huang et al., 2012; Xie et al., 2013). Thus, common and specific brain responses need to be clarified between these conditions for delicate mechanistic understanding of acupuncture.

## Default Mode Network as a Neural Substrate of Acupuncture

Default mode network (DMN) is a recently appreciated brain system, which shows strong activity at rest but deactivates upon externally oriented attention (Buckner et al., 2008; Northoff et al., 2010). Resting state functional magnetic resonance imaging has identified key clusters of human DMN including mPFC, ACC, PCC, orbital frontal cortex, lateral temporal cortex, inferior parietal lobe, retrosplenial cortex and precuneus (Buckner et al., 2008). Simultaneous with signal attenuation in the DMN, a significant signal potentiation in the salience network can be observed (Napadow et al., 2009; Nierhaus et al., 2015), with anterior insula initiating dynamic switching between these intrinsic networks (Bai et al., 2009).

We could note that brain regions within the DMN overlap to a large extent with acupuncture-responsive regions (Chae et al., 2013), which leads to the hypothesis that acupuncture exerts effects through its modulation over the DMN (Otti and Noll-Hussong, 2012; Zhao et al., 2014). In addition to local activation/deactivation, the functional connectivity within and across DMN is also modulated by acupuncture (Dhond et al., 2008; Zyloney et al., 2010; Long et al., 2016; Shi et al., 2016). More importantly, acupuncture-induced deactivation of DMN is stronger than sham acupuncture or tactile stimulation, but attenuated or reversed in direction if sharp pain occurs during acupuncture practice (Hui et al., 2010). In addition, increasing the “dose” of acupuncture, by increasing the number of needles or the intensity of needle stimulation, might induce an enhanced modulation of DMN that persisted even after the termination of acupuncture stimulation (Lin et al., 2016).

Disrupted DMN activities have been observed in various diseases including pain (Dhond et al., 2008; Kucyi et al., 2014; Alshelh et al., 2018), autism (Kennedy and Courchesne, 2008), schizophrenia (Bluhm et al., 2009), Alzheimer’s disease (Sorg et al., 2007), depression (Liston et al., 2014), attention-deficit/hyperactivity disorder (Norman et al., 2017), insomnia (Yu et al., 2018), multiple sclerosis-related fatigue (Jaeger et al., 2018), and posttraumatic stress disorder (Sripada et al., 2012; Akiki et al., 2018). Chronic low back pain is associated with less connectivity within DMN, mainly in the dorsolateral prefrontal cortex, mPFC, ACC and precuneus (Baliki et al., 2008, 2014; Loggia et al., 2013; Ceko et al., 2015; Jiang et al., 2016; Alshelh et al., 2018). Acupuncture reverses these changes almost to the levels seen in healthy controls, and reductions in clinical pain are correlated with increases in DMN connectivity (Li et al., 2014). Similar results are also reported in chronic sciatica patients (Li et al., 2012). In another study on experimental acute low back pain (Shi et al., 2015), pain state induces higher regional homogeneity values in the limbic system and DMN, and acupuncture yields broad deactivation in DMN, consistent with other research as previously described. Apart from pain, acupuncture has also been evaluated in other disorders. In patients with depression, acupuncture induced wide posterior DMN activation (Quah-Smith et al., 2013) and increased functional connectivity between PCC and bilateral ACC (Deng et al., 2016). In stroke patients, enhanced interregional interaction between ACC and PCC, two key DMN hubs, was observed after acupuncture (Zhang et al., 2014). Finally, acupuncture attenuates impaired DMN connectivity seen in patients with Alzheimer disease (Liang et al., 2014).

If DMN is generally affected by acupuncture, we might observe both common and specific modulation of DMN by stimulation at different acupuncture points. Liu et al. performed electro-acupuncture stimulation at three acupuncture points (GB37, BL60, and KI8) and observed consistently interrupted correlation between PCC and ACC, two key nodes of the DMN (Liu et al., 2009b). However, stimulating these three points produced different correlation strength between other nodes in DMN. In addition, visual cortical regions and mPFC are specifically responsive to the stimulation of GB37, whereas KI8 is more associated with activity changes in insula and hippocampus (Liu et al., 2009a). This modulatory pattern is consistent with clinical practice that GB37 is one of the important acupuncture points for eye diseases whereas KI8 is related to gynecological disorders such as menstrual pain. Claunch et al. (2012) examined the specificity and commonality of the brain response to manual acupuncture at LI4, ST36, and LV3, and found clusters of deactivation in the mPFC, medial parietal and medial temporal lobes showing significant convergence of two or all three of the acupuncture points. For differences, LI4 predominated in the pregenual cingulate and hippocampal formation, ST36 predominated in the subgenual cingulate, and LV3 predominated in the posterior hippocampus and PCC. Similar commonality and specificity of brain responses to different acupuncture points, with DMN regions as crucial hubs, are also reported by a series of studies on PC6, PC7, and GB37 (Bai et al., 2010; Ren et al., 2010; Feng et al., 2011).

## A Back-Translational Strategy for Future Research

Despite these correlative observations, direct evidence to causally validate DMN as a neural substrate of acupuncture is lacking: the modulation of DMN by acupuncture could only reflect indirect consequences of other more specific therapeutic effects, or even some insignificant by-products of the stimulation. Additional complexity stems from the fact that DMN changes upon acupuncture could be directly driven by somatosensory afference of acupuncture (i.e., stimulation intensity or de-qi sensation), or indirectly caused by affective or cognitive processes related to the therapeutic effect. Caution should be taken to differentiate between these mechanisms using sham acupuncture methodology. Indeed, it remains a tremendous challenge to causally elucidate brain mechanisms of acupuncture. In the first instance, mechanisms of both physiological and pathological brain networks are still under investigation, before we superimpose acupuncture stimulation above them. For example, the molecular and cellular architecture of DMN is far from clear, despite the discovery of DMN-like networks in laboratory animals (Hayden et al., 2009; Popa et al., 2009; Northoff et al., 2010; Lu et al., 2012; Sforazzini et al., 2014) and some pilot mechanistic findings (Nair et al., 2018; Turchi et al., 2018; Yang et al., 2018). Indeed, mechanisms of acupuncture, pain and other neural processes could not be fully clarified without understanding these network substrates, since the same brain region could participate in distinct processes through different microcircuits (Zheng et al., 2017; Jiang et al., 2018). In addition, acupuncture may exert its effects at multiple levels ranging from local stimulation sites to higher centers in the brain. For example, adenosine locally released in acupuncture sites is sufficient to induce analgesia (Goldman et al., 2010; Takano et al., 2012), in which case brain activity changes may only reflect secondary responses of this peripheral mechanism. However, ACC and other brain regions have a crucial role in at least some forms of acupuncture-induced analgesia (Yi et al., 2011). It is challenging to differentiate between causal brain mechanisms of acupuncture stimulation and secondary responses of peripheral effects.

Despite these challenges, novel techniques, especially those targeting neural circuitries, are becoming available to solve the problem. We propose a back-translational strategy involving several key experimental steps toward scientific verification of brain mechanisms of acupuncture, including the possible role of DMN or other functional networks.

First, the architecture of functional neuronal networks requires elucidation at neuronal and molecular levels. Taking the DMN as an example, the concept of default mode stems from neuroimaging studies primarily based on blood oxygen metabolism, which only indirectly reflects neuronal activities. Recent years have witnessed several intriguing studies linking blood oxygen level-dependent signals with electrophysiological measures of neuronal ensembles, especially high frequency neuronal oscillations in the gamma band (Niessing et al., 2005; Scholvinck et al., 2010). Key brain regions in the DMN revealed from neuroimaging studies in humans could first be confirmed with *in vivo* multi-channel electrophysiological recording in freely behaving animal models, taking advantages of accurate evaluation of cross-regional interactions and their behavioral correlates (Li et al., 2017). Neuronal and molecular substrates of these networks could be further examined with pharmacological and genetic techniques. Special attention may focus on activity- and metabolism-associated molecules such as adenosine triphosphate, adenosine and neurosteroids (Goldman et al., 2010; Zhang et al., 2016, 2017).

With the same techniques, the multi-dimensional brain responses of various acupuncture paradigms could be evaluated at both neuronal network and single cell levels. Such “mapping” studies in animals would complement neuroimaging studies in humans, and form the basis for following causal verification. Computational methods including pattern recognition and machine learning would show their strength in differentiating common and specific brain responses between various stimulating paradigms and to isolate key electrophysiological features.

Finally and most importantly, interventional techniques such as opto- and chemo-genetics are required to causally verify the molecular and neuronal mechanisms of functional networks, the overlying acupuncture effects, and the contribution of different dimensions of brain responses to acupuncture effects. Basal forebrain has been suggested to underlie DMN-like activities in rodents (Nair et al., 2018; Turchi et al., 2018), but causal evidence for this hypothesis is still missing. Similarly, causal contribution of brain activity changes in acupuncture is also lacking. These techniques would finally demonstrate causal contribution of DMN activity changes to acupuncture effects.

With this strategy, one might elucidate brain mechanisms of acupuncture in animal models. This knowledge could then be used to improve future acupuncture studies in humans.

## Author Contributions

MY, DP, and CW designed the perspective. YZ, HZ, TN, and DP reviewed the relevant literature. YZ, HZ, and MY drafted the manuscript. All authors read and confirmed the manuscript.

## Conflict of Interest Statement

The authors declare that the research was conducted in the absence of any commercial or financial relationships that could be construed as a potential conflict of interest.
